# Experience-Based Co-Design to Develop an Injury Prevention Intervention in Skilled Nursing Facilities

**DOI:** 10.1093/geroni/igaa057.853

**Published:** 2020-12-16

**Authors:** Eleanor McConnell, Sarah Berry, Emily Hecker, Laurie Herndon, Cathleen Colon-Emeric

**Affiliations:** 1 Duke University Medical Center, Durham, North Carolina, United States; 2 Hebrew SeniorLife, Marcus Institute for Aging Research, Boston, Massachusetts, United States; 3 Duke University School of Medicine, Durham, North Carolina, United States; 4 Hebrew Seniorlife, Boston, Massachusetts, United States; 5 Duke University, Durham, North Carolina, United States

## Abstract

Experience-based co-design (EBCD) improves clinical effectiveness and safety by incorporating end-user perspectives in the design of clinical interventions. To refine a centralized, multi-component fall-related injury prevention service (IPS) to be tested in skilled nursing facilities (SNFs) in a pragmatic trial, we employed a modified EBCD process. We first conducted in-depth interviews with SNF residents, family members, and staff (n = 28; three facilities in two states) regarding their experiences in falls prevention. We then engaged these and other stakeholders from multiple institutions (n=4) in a day-long co-design workshop with our interdisciplinary research team. Building upon themes drawn from the analysis of interviews, we targeted three intervention components that were refined during the workshop: de-prescribing process, osteoporosis treatment, and educational videoconferences. Key outcomes from the ECBD process included development of strategies to ensure that: (1) residents, families, and SNF staff are involved in communication about residents identified as high risk for fall-related injury, and in related treatment decisions; (2) approaches to monitoring for unintended consequences from the injury prevention plan are clearly understood by direct care staff and are compatible with existing workflow; (3) treatment plan risks and benefits are presented in a manner easily understood by stakeholders; and (4) staff education conferences build trust with the IPS nurse and provide direct care staff with support and advice about challenging cases. EBCD is a feasible approach to strengthen intervention development in SNFs and can lead to testable new ideas for protocol refinement to address diverse stakeholder perspectives.

